# A Comparative Study on the Growth Performance and Gut Microbial Composition of Duroc and Yorkshire Boars

**DOI:** 10.3390/genes14091726

**Published:** 2023-08-29

**Authors:** Junhua Du, Mailin Gan, Zhongwei Xie, Gao Du, Yi Luo, Bin Liu, Kangping Zhu, Lei Chen, Ye Zhao, Lili Niu, Yan Wang, Jingyong Wang, Li Zhu, Linyuan Shen

**Affiliations:** 1 Farm Animal Genetic Resources Exploration and Innovation Key Laboratory of Sichuan Province, Sichuan Agricultural University, Chengdu 611130, China; 2021302172@stu.sicau.edu.cn (J.D.); ganmailin@sicau.edu.cn (M.G.); 2021302137@stu.sicau.edu.cn (Z.X.); 15528117725@163.com (G.D.); chenlei815918@sicau.edu.cn (L.C.); zhye@sicau.edu.cn (Y.Z.); niulili@sicau.edu.cn (L.N.); 14916@sicau.edu.cn (Y.W.); zhuli@sicau.edu.cn (L.Z.); 2 Key Laboratory of Livestock and Poultry Multi-Omics, Ministry of Agriculture and Rural Affairs, College of Animal and Technology, Sichuan Agricultural University, Chengdu 611130, China; 3 Sichuan Dekon Livestock Foodstuff Group, Chengdu 610200, China; luoyi670@163.com (Y.L.); 13018240830@163.com (B.L.); guchebcunerzu@163.com (K.Z.); 4 Chongqing Academy of Animal Science, Chongqing 402460, China; kingyou@vip.sina.com

**Keywords:** boar, breed, growth performance, gut microbiota

## Abstract

The intestinal microbiota is required for maintaining the development and health of the host. However, the gut microbiota contributing to the regulation of the growth performance and health of Duroc and Yorkshire boars remains largely unknown. In this study, we first evaluated the difference in the growth performance between Duroc and Yorkshire boars when their body weight reached 100 kg. Relative to Duroc boars, Yorkshire boars weighed 100 kg at a younger age and exhibited a significantly lower backfat thickness and eye muscle area. Microbial analysis of the fecal samples revealed a marked difference in gut microbiota composition between the two pig models and remarkably increased α-diversity in Yorkshire boars compared to Duroc boars. Further analysis indicated that Bacteroidota, Prevotellaceae, and Ruminococcaceae might be associated with the growth performance and lean meat rate of Yorkshire boars. Taken together, these results highlight that Yorkshire boars exhibit a faster growth rate and higher lean meat rate compared to Duroc boars, and these differences may be attributed to the influence of the gut microbiota, thereby providing valuable insight into optimizing pig breeding systems and selecting terminal paternal sires to enhance overall productivity and quality.

## 1. Introduction

Microbial is a comprehensive term encompassing all microorganisms that inhabit the bodies and surfaces of host animals. Notably, the intestinal tract houses the greatest diversity and abundance of microbial species, predominantly comprising archaea, fungi, bacteria, protozoa, and various other microorganisms [1]. The intestine serves as the primary site for food digestion and nutrient absorption and stands as the body’s largest immune organ [2]. As such, the intestinal microbial community assumes a pivotal role in the host’s processes of nutrient digestion, energy metabolism, and immune regulation [3,4,5]. An increasing body of research indicates a significant correlation between alterations in the composition and functionality of the intestinal microbiota and the health and production performance of pigs. Therefore, comprehending and mitigating the factors that influence the gut microbiota’s composition and function become imperative in the context of pig production [6,7,8].

Numerous factors exert an influence on the composition and function of intestinal microbial communities, including environmental management, feed composition, additive utilization, and genetic background. Although [9] emphasized the role of diet as the primary factor influencing the composition of intestinal microbial communities, a growing number of research works has unveiled that host genetic factors also have a certain impact on the composition of intestinal microorganisms [10,11,12]. Cheng et al. [13] indicated that, within a consistent feeding environment and uniform dietary conditions, the quantity of microorganisms present in the feces of Lantang pigs surpassed that of Duroc pigs. Moreover, Yang et al. [10] analyzed the intestinal microbial communities across eight pig breeds and revealed a strong similarity in gut microbial communities among foreign pig breeds such as Large White and Duroc pigs, while local Chinese pig breeds such as Bama Xiang pigs, Erhualian pigs, and Meishan pigs displayed certain degrees of similarity. Duroc pigs extensively serve as the terminal male parent in commercial pig hybrid lines, owing to their notable advantages such as rapid growth, exceptional feed utilization, and favorable carcass quality [14]. In recent years, concerns have arisen due to the decreasing population of Duroc boars, less-favorable reproductive traits, and sluggish growth and development (including reduced stress resistance) under challenging breeding conditions [15]. In response to market demands, international breeding companies have developed paternal Yorkshire boars with comparable traits to Duroc boars to serve as terminal male parents. Despite the significance of this shift, there is currently a lack of studies that directly compare the growth performance and intestinal microbial composition between Duroc and Yorkshire boars. Therefore, it becomes crucial for the pig industry to discern the differences in the growth performance and intestinal microbial diversity exhibited by these two boar breeds. Therefore, we mainly analyzed the age, backfat thickness, eye muscle area, and composition of the gut microbiota communities of Duroc and Yorkshire boars under the same feeding conditions, aiming to show the difference in their growth rates and microbiota compositions. More importantly, we provide valuable insights into optimizing pig breeding systems and selecting terminal paternal parents to enhance overall productivity and quality.

## 2. Materials and Methods

### 2.1. Animals, Experimental Design, and Sample Collection

A total of 148 Duroc boars (DD) and 69 Yorkshire boars (PY) were in included in the study, all of which were born from the same batch. Consistency was maintained in their feeding regimen, water provision, and environment conditions throughout the entire study duration. The pigs’ basal diet’s composition and nutrient levels during the growth stages were sourced from Zigong Dekang Agriculture and Animal Husbandry Technology Co., Ltd. (Sichuan, China) as detailed in Supplementary Table S1. Upon all boars reaching a body weight of 100 kg, their backfat thickness and eye muscle area were quantified using B-mode real-time ultrasound technology. The backfat thickness was assessed by measuring the vertical distance from the skin on the back to the longissimus dorsi membrane at a point 5 cm away from the penultimate 3rd and 4th ribs on the midline of the back. The eye muscle area was quantified at the last rib using vernier calipers. Fecal samples were freshly collected from the rectal region of individual pigs at approximately 60 days of age using a gloved finger. Subsequently, individual fecal samples were immediately placed into a sterile Whirl-Pak bag and stored on dry ice. All samples were transported to the laboratory and stored at −80 °C for further processing. In order to avoid the contamination of the samples, the gloves were changed between pigs.

All animals were obtained from a reputable pig-breeding enterprise in Sichuan Province, China. All experimental procedures were duly approved by the Animal Ethical and Welfare Committee of Sichuan Agricultural University, Chengdu, China (Approval Number 2021302137).

### 2.2. 16S rDNA Gene Sequencing

Fecal samples were subjected to DNA extraction using the CTAB method, following the manufacturer’s protocols. The purity and concentration of the obtained DNA were assessed using agarose gel electrophoresis. Subsequently, the genomic DNA was suitably diluted for PCR amplification. The 341F (CCTAYGGGRBGCASCAG) and 806R (GGACTACNNGGGTATCTAAT) amplification primers were used to target the V3-V4 region of the bacteria during PCR amplification. The resulting PCR products were verified for presence using 2% agarose gel electrophoresis. For library construction, the NEBNext^®^ Ultra™ II DNA Library Prep Kit (NEB) was employed. The ensuing library was quantified through the Qubit and Q-PCR methodologies. Upon successful library validation, the NovaSeq6000 (Illumina San Diego, CA, USA) platform was harnessed for sequencing purposes.

### 2.3. Bioinformatics Analysis of Sequencing Data

The original sequence underwent filtration using FASTP (https://github.com/OpenGene/fastp, accessed on 10 June 2023) [16]. To obtain the final valid data, the Tags quality control process of QIIME [17,18] was followed. The Uparse algorithm (Uparse v7.0.1001, http://www.drive5.com/uparse/, accessed on 10 June 2023) [19] was utilized to cluster all Effective Tags from the samples, resulting in the sequence being grouped into operational taxonomic units (OTUs) with a default 97% identity threshold. The α-diversity indices, including Shannon, Observed, and Ace, along with the β-diversity metrics, PCoA and NMDS, were computed using the Qiime 1.9.1 software. These analyses were employed to assess the richness and distinctive distribution of the microbiota community, respectively. Furthermore, the dilution curve was utilized to gauge the adequacy of the sequencing quantity. To identify the distinct species between different groups, the LEfSe 1.1 software was employed for the analysis, which helped determine the significantly different species. Additionally, the linear discriminant score (LDA Score) was utilized to quantify the impact of different species on the dissimilarity observed between groups. Finally, in order to discern the variation and shifts in the metabolic pathways of functional genes within the microbial community across the two groups, the FAAPROTAX database, widely used in microbial diversity analysis, was employed to annotate the microbial community.

### 2.4. Statistical Analysis

The age, backfat thickness, eye muscle area, and relative abundance of the fecal microbes in the boars were analyzed using the SPSS 22.0 software. Differences between the mean values were assessed using the independent sample *t*-test, with significance set at a *p*-value of less than 0.05.

## 3. Results

### 3.1. Effect of Different Breeds on Growth Performance of Boars

To investigate the impact of different breeds on the growth performance of boars, we compared the age, backfat, and muscle area of boars weighing 100 kg between the two breeds. As shown in Table 1, the boars in the PY group exhibited a significant reduction in age, backfat, and eye muscle area when compared to the DD group. The age result may suggest that the growth rate of the PY group was higher in comparison to the DD group.

### 3.2. Diversity Analysis of Gut Microbiota

**Figure 1:**
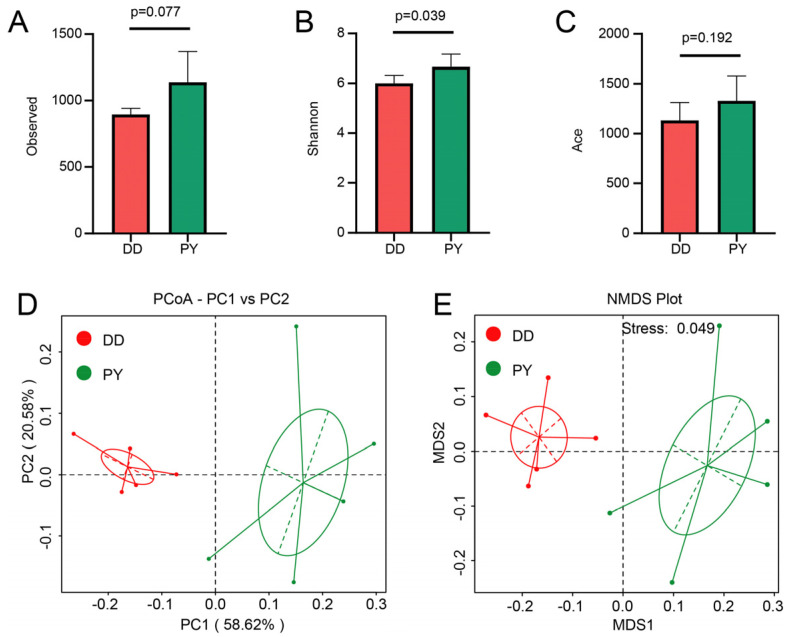
Diversity analysis of the gut microbiota. (**A**–**C**) Alpha diversity analysis of Observed, Shannon, and Ace indexes. (**D**) Principal coordinates analysis (PCoA). (**E**) Non-metric multi-dimensional scaling (NMDS).

Numerous pieces of evidence have brought to light the potential involvement of the gut microbiota in mediating the impact of nutrients on growth performance. To elucidate the regulatory mechanisms by which different breeds affect the growth performance of boars, we analyzed the gut microbiota using 16S rDNA gene sequencing on fecal samples collected from both the DD and PY groups. Following the quality control, a total of 2847 valid operational taxonomic units (OTUs) were generated for each sample, which were then clustered at 97% similarity for subsequent analysis. Supplementary Figure S1 illustrates the analysis of the dilution curve, indicating that the sequencing depth adequately covered rare new phylotypes and captured a substantial portion of the overall diversity. To gain insight into the alterations of the gut microbiota in boars between the two breeds, we assessed the Alpha diversity using specific indices such as richness (Observed and Ace) and evenness (Shannon). As shown in Figure 1A–C, the PY group displayed a higher evenness of microbiota, indicated by the increased Shannon index, compared to the DD group. Additionally, the PY group showed a noticeable upward trend in the richness of the microbiota, as evidenced by the Observed index, when compared to the DD group. Subsequently, we analyzed the overall changes in the microbial community of the boars between the two breeds by using both principal coordinates analysis (PCoA) and non-metric multi-dimensional scaling (NMDS). According to the PCoA result, a noteworthy distinction between the two breeds was evident (Figure 1D). Notably, PC1 and PC2 accounted for 58.62% and 20.58% of the total variance, respectively (Figure 1D). In parallel, the NMDS analysis further indicated that the bacterial community’s composition and structure exhibited substantial disparities among the two breeds (Figure 1E).

### 3.3. Altered Gut Microbiota at Different Taxonomic Levels

**Figure 2:**
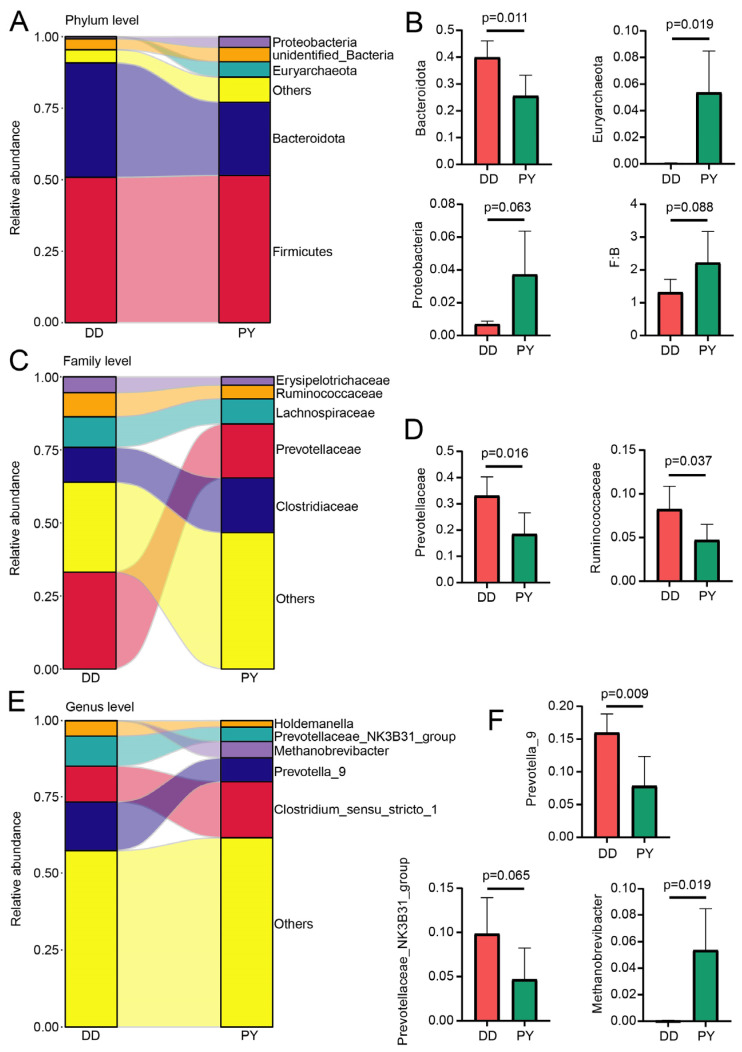
Altered gut microbiota at different taxonomic levels. (**A**,**C**,**E**) Microbial composition of the feces at the phylum (**A**), family (**C**), and genus (**E**) levels. (**B**) The relative abundances of differential phyla were compared between the groups. (**D**) The relative abundances of differential families were compared between the groups. (**F**) The relative abundances of differential genera were compared between the groups.

In line with the patterns revealed by the PCoA and NMDS results, the proportions of bacteria in the two breeds exhibited variations at different taxonomic levels. At the phylum level, the prevailing bacteria observed in all groups were Firmicutes, Bacteroidota, Euryarchaeota, unidentified_Bacteria, and Proteobacteria, accounting for more than 95% of the total composition (Figure 2A). The PY group demonstrated a decrease in Bacteroidota and a concurrent rise in Euryarchaeota compared with the DD group (Figure 2B). In addition, the relative abundance of Proteobacteria in the PY group exhibited a higher trend when compared to the DD group (Figure 2B). Considering that a substantially higher abundance ratio of Firmicutes to Bacteroidota (F:B) is commonly considered a marker of metabolic health, we meticulously examined the F:B ratio in both groups. The F:B ratio displayed a significant increase from 1.32 in the DD group to 2.22 in the PY group (Figure 2B), implying an improvement in the metabolism of the boars in the PY group. At the family level, the relative abundance of Prevotellaceae and Runminococcaceae in the PY group significantly decreased when compared to the DD group (Figure 2C,D). Subsequently, further analysis at the genus level revealed significant differences between the two groups in the relative abundance of two genera of bacteria. The relative abundance of Methanobrevibacter (from 0.04% to 5.37%) significantly increased in the PY pigs compared with that in the control group, whereas the relative abundance of Prevotella significantly decreased, and there was a noticeable declining trend in the prevotellaceae_NK3B31_group (Figure 2E,F).

**Figure 3:**
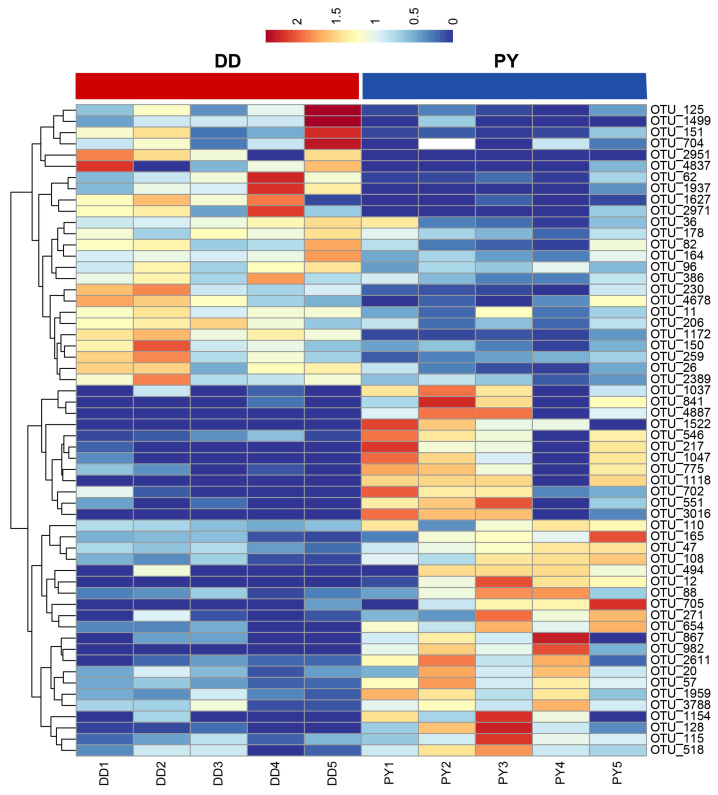
Heatmap showing the relative abundance of differential bacterial OTUs.

To more deeply understand the effect of different breeds on the gut microbiota in the boars, we further identified a total of 59 OTUs that exhibited significant alterations. Among these, 34 OTUs demonstrated an increase, while 25 OTUs showed a decrease in relative abundance between the groups (Figure 3). Taken together, these results suggest that the boars of the PY breed had a distinctly different gut microbiota community compared to the DD breed.

### 3.4. Screening the Fecal Microbiota Biomarkers

**Figure 4:**
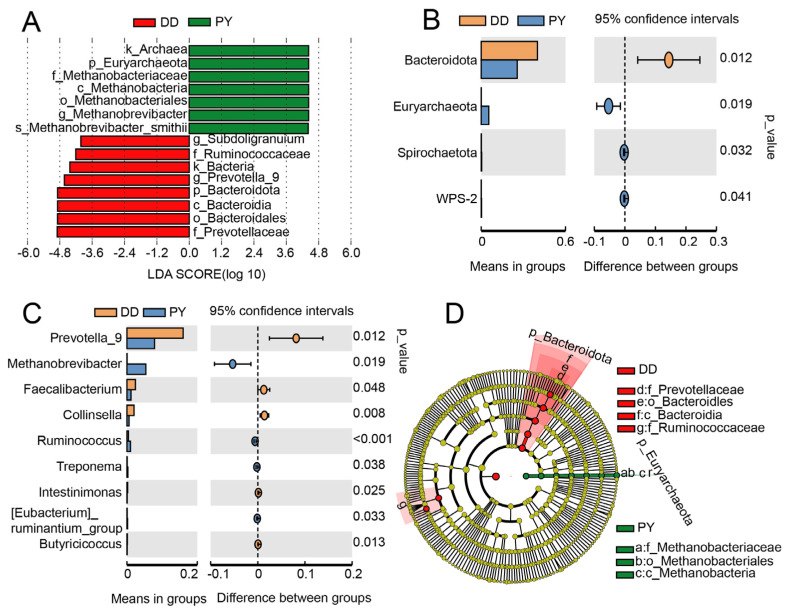
Screening the fecal microbiota biomarkers. (**A**) Histogram of the results of LEfSe among the PY group and the control group and their respective effect sizes. (**B**) *t*-test analysis at the phylum level. (**C**) *t*-test analysis at the genus level. (**D**) Cladogram showing the taxonomic representation of the differences among the PY group and the control group.

An LEfSe analysis was employed to identify fecal biomarkers associated with the two breeds, based on the significantly different bacteria screened at the phylum, family, and genus levels. A total of 15 biomarkers exhibited significant differences in the relative abundance between the DD breed and the PY breed at different taxonomic levels. Candidate biomarkers in the PY breed included k_Archaea, p_Euryarchaeota, f_Methanobacteria, c_Methanobacteria, o_Methanobacteriales, g_Methanobrevibacter, and s_Methanobrevibacter_smithii, and those in the DD breed included g_Subdoligranuium, f_Ruminococcaceae, k_Bacteria, g_Prevotella_9, p_Bacteroidota, c_Bacteroidia, o_Bacteroidales, and f_Prevotellaceae (Figure 4A). To further undertake a more-comprehensive comparison of the relative abundance of the candidate markers between the two groups, we primarily used t-test analysis at the phylum and genus level. Consistent with the results depicted in Figure 2, the relative abundance of p_Bacteroidota and g_Prevotella_9 in the PY group exhibited a notable reduction, while the relative abundance of p_Euryarchaeota and g_Methanobrevibacter demonstrated a marked increase in comparison to the DD group (Figure 4B,C). It was noteworthy that, at the phylum level, Bacteroidota and Euryarchaeota emerged as the most-differentially abundant taxa in the PY breed (Figure 4D). Therefore, these pivotal phylotypes are likely to contribute to the observed variations in the microbiota composition among different groups.

### 3.5. FAPROTAX Predictions of Gut Microbe Functions

**Figure 5:**
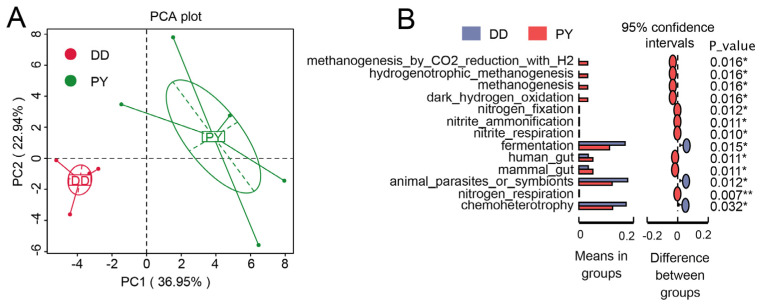
Prediction of metabolic pathways regulated by fecal microbes. (**A**) Principal component analysis. (**B**) *t*-test analysis. * *p* < 0.05; ** *p* < 0.01.

To gain more insight into the effect of different breeds on the gut microbiota, we investigated the potential function of the gut microbiota by using FAPROTAX analysis. Predictive function richness was used to generate a principal component analysis (PCA) plot, which clearly showed separate clustering of samples from the PY group and control group (Figure 5A). Through the implementation of FAPROTAX 1.0 software, we identified 13 metabolic pathways with the most-significant differences. Among these, 10 metabolic pathways significantly increased in the PY breed compared with the DD breed, including methanogenesis_by_CO2_reduction_with_H2, hydrogenotrophic_methanogenesis, methanogenesis, dark_hydrogen_oxidation, nitrogen_fixation, nitrite_ammonification, nitrite_respiration, hunman_gut, mammal_gut, and nitrogen_respiration (Figure 5B), while the fermentation, animal_parasites_or_symbionts, and chemoheterotrophy pathways significantly decreased.

## 4. Discussion

In the pig industry, there is a strong emphasis on breeding varieties with exceptional production performance, rapid growth, and high feed conversion rates. Therefore, the breeding of boars with outstanding production performance to serve as terminal male parents holds immense significance for the advancement of the pig industry. A body weight of 100 kg commonly serves as an indicator of the growth rate and feed efficiency of tested pigs [20], a threshold that also aligns with the optimal slaughter weight for lean pigs. In this study, we observed the age, backfat thickness, and eye muscle area of boars between the DD and PY groups when they weighted 100 kg. The PY group achieved the 100 kg weight at a younger age and exhibited a relatively thinner backfat thickness compared to the DD group. In accordance with Fan’s findings [21], our study also demonstrated that Yorkshire boars exhibit faster growth. In addition, research has demonstrated that a decrease in the pig’s backfat thickness is associated with an increase in the eye muscle area, thereby indicating a higher lean meat percentage [22]. However, our results revealed that Yorkshire boars displayed reduced backfat thickness and eye muscle area, a pattern that deviates from the trends observed in other studies. Furthermore, Guo showed that Duroc pigs had a significantly higher lean meat rate compared to Yorkshire pigs [23]. The underlying causes for this inconsistency necessitate further investigation.

The composition of the gut microbiota plays a pivotal role in influencing various aspects of the host, such as growth, metabolism, reproduction, and even meat quality. Given that the intestinal development of pigs commonly attains a relatively mature state during the fattening phase, the composition of the intestinal microbial community tends to stabilize [9]. It is worth noting that the composition of the intestinal microbiota in pigs can vary between different breeds, and the genetic factors of the host can significantly influence the composition of these microorganisms [24,25,26]. According to the α-diversity analysis, the PY group displayed higher richness and evenness of bacteria, indicating a more-intricate and -stable composition of the intestinal flora and a stronger ability to withstand external interference. Moreover, the PCoA and NMDS results demonstrated a clear separation between the two groups, signifying significant differences in bacterial composition between the DD and the PY breeds.

The Firmicutes and Bacteroidota groups make up 80–90% of the proportion of pig intestinal microbiota [27,28,29], but their proportions differ across various pig breeds. For instance, in Jinhua pigs, Firmicutes (70.4%) and Bacteroidota (14.4%) constitute 84.8% of the intestinal microbiota [30]. Conversely, an analysis of Duroc, Yorkshire, and Landrace pigs indicated that Firmicutes accounted for only 40–45%, while Bacteroidota accounted for 47–57% [31]. At the phylum level, we found that the highest proportion was Firmicutes and Bacteroidota, making up 85–90%, which is consistent with findings from previous research. Reference [32] indicated the intestinal microorganisms of pigs at different stages and found that, as pigs age, the proportion of Bacteroidota decreases, leading to incomplete carbohydrate decomposition and accumulation in the body, resulting in weight gain. Therefore, the Firmicutes/Bacteroidetes ratio can be a marker of obesity [33]. In our study, the F:B ratio displayed a significant increase from 1.32 in the DD group to 2.22 in the PY group, which may serve as one of the crucial factors contributing to the faster growth observed in Yorkshire boars. The Prevotellaceae NK3B31 group and Prevotella_9, which belong to the genus Prevotella, also exhibit a growth-promoting effect because they can reduce inflammation by decreasing intestinal permeability [7]. However, our study indicated that the relative abundance of the Prevotellaceae NK3B31 group and Prevotella_9 in the PY group significantly decreased compared to the control group, which may be due to the small number of samples. In addition, the top three core microorganisms at the family level, including Prevotellaceae, Ruminococcaceae, as well as and Lactobacillaceae are closely related to lipid metabolism in pigs [34]. Reference [35] indicated that the Prevotellaceae family plays a crucial role in regulating pig fat deposition, acting as a core microorganism. Its abundance has been found to be negatively correlated with carcass lean meat percentage, implying its potential influence on the fat content in pigs. In our study, we noted a significant reduction in the relative abundance of Prevotellaceae and Ruminococcaceae, which could potentially account for the lower backfat thickness observed in Yorkshire boars. Finally, we analyzed the KEGG pathway of the differential microorganisms between the paternal Yorkshire and Duroc boars and predicted their functions. Our findings revealed that the functional clustering could effectively distinguish between the two breeds, indicating a significant difference in the primary functional composition of the intestinal microorganisms. Animal_parasites_or_symbionts, chemoheterotrophy, and fermentation were the most-abundant pathways in both paternal Yorkshire and Duroc boars, with the Duroc boars exhibiting a significantly higher abundance, while these three functions are related to microbial metabolism [36,37].

## 5. Conclusions

In this study, Yorkshire boars achieved a 100 kg body weight at a younger age and exhibited a slimmer backfat thickness in comparison to Duroc boars. These findings indicated that Yorkshire boars have accelerated growth rates and a higher proportion of lean tissue. Moreover, these observed phenotypic disparities might be attributed to the influence of gut microbiota composition, as suggested by our gut microbiota analysis. Nonetheless, it is important to acknowledge that our analysis of the gut microbiota was based on a relatively small number of pig samples. To enhance the robustness and precision of our research outcomes, future investigations should consider expanding the sample size to encompass a more-representative range of subjects.

## Figures and Tables

**Table 1:** The comparison of age, backfat, and eye muscle area of boars weighing 100 kg between the two groups.

Item	Group		*p*-Value
	DD (148)	PY (69)	
Age, day	164.95 ± 9.11	145.58 ± 8.86	<0.01
Backfat, mm	6.77 ± 2.01	5.30 ± 1.55	<0.01
Eye muscle area, cm^2^	64.13 ± 8.43	54.98 ± 4.42	<0.01

## Data Availability

Not applicable.

## Supplementary Materials

The following Supporting Information can be downloaded at: https://www.mdpi.com/article/10.3390/genes14091726/s1, Figure S1: The dilution curve of all samples; Table S1: The composition and nutrient levels of the basal diet.
