# Supplementary material for: A Comparative Study on the Growth Performance and Gut Microbial Composition of Duroc and Yorkshire Boars
Source: Genes (Basel). 2023 Aug 29;14(9):1726. doi: 10.3390/genes14091726 (PMC10531244; doi:10.3390/genes14091726)
Supplement: Supplementary file 1 [file genes-14-01726-s001.zip › Supplementary Table 1.pdf]

Supplementary Table S1. The composition and nutrient levels of the basal diet.

Feed ingredient	Nutrient levels	Content (%)
Corn	Crude protein	12.5
Wheat	Crude fiber	⩽8.0
Soybean meal	Crude ash	⩽8.0
Soybean oil	Calcium	0.4-1.2
Wheat flour	Total phosphorus	⩾0.35
Stone powder	Chloride	0.3-1.0
Calcium hydrogen phosphate	Moisture	⩽14.0
Sodium chloride	Lysine	0.6
Amino acids		
Vitamins (A, D, and E)		
Phytase		
