# Supplementary figures and images for: A Comparative Study on the Growth Performance and Gut Microbial Composition of Duroc and Yorkshire Boars
Source: Genes (Basel). 2023 Aug 29;14(9):1726. doi: 10.3390/genes14091726 (PMC10531244; doi:10.3390/genes14091726)

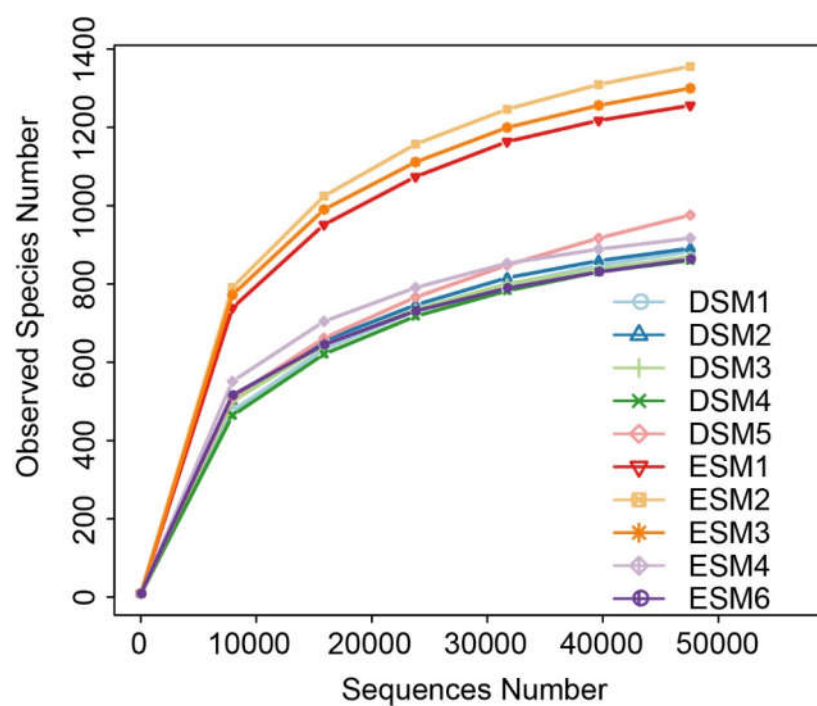

Supplementary Figure S1. The dilution curve of all samples.

Supplement: Supplementary file 1 [file genes-14-01726-s001.zip › Supplementary Figure 1.pdf]
